# A Single Nucleotide Polymorphism in the *RASGRF2* Gene Is Associated with Alcoholic Liver Cirrhosis in Men

**DOI:** 10.1371/journal.pone.0168685

**Published:** 2016-12-19

**Authors:** Ignacio Novo-Veleiro, Clara Cieza-Borrella, Isabel Pastor, Antonio-Javier Chamorro, Francisco-Javier Laso, Rogelio González-Sarmiento, Miguel Marcos

**Affiliations:** 1 Department of Internal Medicine, University Hospital of Santiago de Compostela, A Coruña, Spain; 2 Molecular Medicine Unit, Department of Medicine, University of Salamanca, Salamanca, Spain; 3 Institute of Biomedical Research of Salamanca-IBSAL, Salamanca, Spain; 4 Alcoholism Unit, Department of Internal Medicine, University Hospital of Salamanca, Salamanca, Spain; Medizinische Fakultat der RWTH Aachen, GERMANY

## Abstract

**Background:**

Genetic polymorphisms in the *RAS* gene family are associated with different diseases, which may include alcohol-related disorders. Previous studies showed an association of the allelic variant rs26907 in *RASGRF2* gene with higher alcohol intake. Additionally, the rs61764370 polymorphism in the *KRAS* gene is located in a binding site for the *let-7* micro-RNA family, which is potentially involved in alcohol-induced inflammation. Therefore, this study was designed to explore the association between these two polymorphisms and susceptibility to alcoholism or alcoholic liver disease (ALD).

**Methods:**

We enrolled 301 male alcoholic patients and 156 healthy male volunteers in this study. Polymorphisms were genotyped by using TaqMan^®^ PCR assays for allelic discrimination. Allelic and genotypic frequencies were compared between the two groups. Logistic regression analysis was performed to analyze the inheritance model.

**Results:**

The A allele of the *RASGRF2* polymorphism (rs26907) was significantly more prevalent among alcoholic patients with cirrhosis (23.2%) compared to alcoholic patients without ALD (14.2%). This difference remained significant in the group of patients with alcohol dependence (28.8% vs. 14.3%) but not in those with alcohol abuse (15.1% vs. 14.4%). Multivariable logistic regression analysis showed that the A allele of this polymorphism (AA or GA genotype) was associated with alcoholic cirrhosis both in the total group of alcoholics (odds ratio [OR]: 2.33, 95% confidence interval [CI]: 1.32–4.11; *P* = 0.002) and in the group of patients with alcohol dependence (OR: 3.1, 95% CI: 1.50–6.20; *P* = 0.001). Allelic distributions of the *KRAS* polymorphism (rs61764370) did not differ between the groups.

**Conclusions:**

To our knowledge, this genetic association study represents the first to show an association of the *RASGRF2* G>A (rs26907) polymorphism with ALD in men, particularly in the subgroup of patients with AD. The findings suggest the potential relevance of the *RAS* gene family in alcoholism and ALD.

## Introduction

Genetic predisposition plays a major role in susceptibility to alcoholism [1] or ethanol-induced organ damage [2]. Many association studies have been conducted to identify specific genetic variants in these conditions. Most studies of alcohol use disorders and alcohol dependence (AD) have focused on candidate genes involved in brain reward pathways [3]. Meanwhile, genetic studies related to alcoholic liver disease (ALD) have centered on genes coding for detoxifying enzymes [4,5] or inflammatory response mediators [6,7]. A recent meta-analysis of genome-wide association studies (GWAS) provided new insights into genes associated with AD [8]. Nevertheless, the complete profile of genetic variants underlying susceptibility to alcohol use disorders or ALD remains unknown.

In this setting, genetic polymorphisms of members of the *RAS* gene family have been associated with alcohol-related problems, through their roles in several biological processes, such as neurotransmission and inflammation [9,10]. Specifically, the single nucleotide polymorphism (SNP) rs26907 in the Ras-specific guanine nucleotide-releasing factor 2 gene (*RASGRF2*) was linked to higher alcohol consumption [11] and variability in alcohol-induced reward response. These findings suggest a potential effect of this SNP on alcoholism susceptibility [12]. This is reinforced by the role of Ras protein, which is regulated by RasGRF2, in synaptic transmission [13]. In addition, RasGRF2 has an implied role in inflammatory pathways, through its activation of mitogen-activated protein kinases (MAPKs) and extracellular signal-regulated kinases (ERKs) [14,15]. This observation suggests a link of this protein with ethanol-induced inflammation and, thus, with other alcohol-related problems.

Other polymorphisms within the *RAS* gene family have been associated with human diseases [16]. The rs61764370 polymorphism of the GTPase *KRAS* gene is located in a micro-RNA (miRNA) binding site for members of the *let-7* miRNA family. Possession of the G allele hampers binding of *let-7* and has been associated with different diseases [17]. This polymorphism may also be relevant in alcoholism, as one GWAS showed a relationship between alcohol craving and K-ras activity in an animal model [18]. In addition, *let-7*, which is overexpressed in alcoholic brains, has been associated with the alcohol-triggered inflammatory response [19,20]. The regulatory functions of *let-7* miRNA family members also affect hepatic stellate cell activation [19] and liver disease [21], which may imply a function in ALD development.

In light of these data, the present study was designed to analyze the potential relationships between alcohol-related problems and the SNPs *KRAS* 3377 T>G (rs61764370) and *RASGRF2* 113808 G>A (rs26907).

## Materials and Methods

### Patients and controls

For this study, we enrolled 301 male alcoholic patients from the Alcoholism Unit of the University Hospital of Salamanca (Spain), with an average age of 52.2 years (standard deviation [SD] = 12.5 years; range = 24–80). All patients reported a daily alcohol consumption of more than 100 g for at least 10 years. According to the Diagnostic and Statistical Manual of Mental Disorders, Fourth Edition (DSM-IV) [22] criteria, which were valid during the study period and implemented through the use of semistructured interviews conducted by trained staff members, 189 patients were diagnosed of AD and 112 patients of alcohol abuse (AA). Patients with addictions to other drugs (apart from nicotine) or with major Axis I disorders (i.e. schizophrenia, mood disorders, or major anxiety disorders), established by a comprehensive psychiatric examination, were excluded.

As we previously described for our cohort [23,24], the diagnosis of alcoholic cirrhosis (AC) in 103 of the 301 alcoholic patients was established by liver biopsy in 93 patients or by the presence of clinical, analytical, endoscopic, and/or radiological criteria in the other 10 patients, in whom biopsy was contraindicated. The other 198 alcoholic patients were not considered to have ALD according to clinical, analytical, and radiological criteria. All patients had negative results on hepatitis C and hepatitis B tests. Other causes of liver disease (e.g., haemochromatosis or autoimmune liver disease) were excluded by appropriate tests. Within cirrhotic patients, 43 (41.7%) had AA and 60 (58.3%) AD; these percentages were 34.8% and 65.2%, respectively, in the group of alcoholic patients without ALD.

A control group of 156 healthy male volunteers with mean age of 47 years (SD = 19.6 years, range: 21–88) was also enrolled. Participants in the control group consumed less than 10 g of ethanol per day. Neither the control group participants nor their first- or second-degree relatives had a history of AA or AD.

All patients and controls were at least third-generation Spaniards, born in northwestern Spain. All participants provided informed written consent for study participation. The study was carried out with approval from the Ethics Committee of the University Hospital of Salamanca.

### Genetic analysis

Genomic DNA was extracted from nucleated peripheral blood cells by using standard proteinase K digestion, phenol-chloroform extraction, and ethanol precipitation. Samples were stored at -20°C until use. Samples from healthy controls, alcoholic patients with AC (AC patients), and alcoholic patients without ALD (AWLD patients) were mixed on PCR plates to ensure simultaneous detection and analysis in a blinded fashion. The rs61764370 and rs26907 polymorphisms were genotyped by using TaqMan MGB^®^ assays for allelic discrimination with the StepOnePlus^®^ System (Applied Biosystems, Foster City, CA, USA), according to the manufacturers’ protocols. Genotyping rates were 99.3% among cases and 99.4% among controls for KRAS 3377 T>G (rs61764370) polymorphism and 95.7% among cases and 97.4% among controls for RASGRF2 113808 G>A (rs26907) polymorphism. The consistency of genotyping process was checked by genotyping 25% of the samples in duplicate, showing consistent results.

### Statistical analysis

As previously described [23,24], differences in allelic and genotypic frequencies between groups were compared by the χ^2^ xor Fisher’s exact test, as appropriate (when the expected frequency value was < 5). Deviations from Hardy–Weinberg equilibrium in genotype frequencies among controls were assessed by the χ^2^ test. A *P* value < 0.05 was considered significant. Alcoholic patients were compared to controls, and AC patients were compared to AWLD patients.

The independent contribution of genetic markers to the phenotype was tested by using regression logistic analysis, in order to evaluate recessive versus dominant inheritance of specific alleles. Considering the analysis of the *RASGRF2* G>A polymorphism, arbitrary effects for heterozygous individuals were included in the general model. The three possible genotypes (GG, AG, and AA) were denoted by three dummy variables (0, 1, and 2). The dominant model was based on the hypothesis that the AG and AA genotypes contributed equally to the risk of AC, and the genotypes were coded as follows: GG = 0 and AG and AA = 1. The recessive model was based on the hypothesis that only the AA genotype contributed to the risk of AC, and the genotypes were coded as follows: AG and GG = 0 and AA = 1. Akaike's information criterion (AIC), which combines goodness of fit with the number of parameters, was used to select the best model. The model with the lowest AIC value was considered to have the best fit [25]. Strength of association was calculated with the odds ratio (OR) and 95% confidence interval (CI). A *P* value < 0.05 was regarded as significant. All statistical analyses were performed by using SPSS version 20.0 (IBM SPSS Statistics).

The statistical power of this study to detect a polymorphism conferring an OR of 2 for the presence of alcoholism or AC was calculated by the Power and Sample Size Calculations software, version 3.0.43 (available at http://biostat.mc.vanderbilt.edu/twiki/bin/view/Main/PowerSampleSize) [26]. For alcoholism, assuming an α of 0.05 and a prevalence of the less-frequent allele among controls of 0.2, the statistical power was 81% for the total study population (alcoholic patients, n = 301; healthy controls, n = 156). For AC, with the same assumptions, the statistical power was 71.4% for patients with alcoholism (alcoholics with AC, n = 103; alcoholics without liver disease, n = 198). Post-hoc power calculation was performed with ClinCalc post-hoc power calculator (available at http://clincalc.com/stats/power.aspx) [27].

## Results

Genotypic and allelic distributions of both polymorphisms among alcoholic patients and healthy controls are shown in Tables 1 and 2 and supplementary Table 1. The genotypic distribution among healthy controls was similar to that previously reported among healthy Caucasians [28–30]. No significant deviation from Hardy–Weinberg equilibrium was found.

**Table 1:** Genotypic and allelic frequencies of the *KRAS* and *RASGRF2* polymorphisms in alcoholic patients vs. controls and in patients with alcoholic cirrhosis vs. patients without alcoholic liver disease.

		Alcoholic patients vs. controls		*P*	AC vs. AWLD patients		*P*
		Patients (n = 301)	Controls (n = 156)		AC (n = 103)	AWLD (n = 198)	
*KRAS* rs61764370	TT	232 (77.6)	110 (71)	0.280	84 (81.6)	148 (75.5)	0.063
	GT	63 (27.7)	43 (27.7)		16 (15.5)	47 (24)	
	GG	4 (1.3)	2 (1.3)		3 (2.9)	1 (0.5)	
	MAF (G)	71 (11.9)	47 (15.2)	0.121	22 (10.7)	49 (12.5)	0.234
*RASGRF2* rs26907	GG	196 (68.1)	109 (71.7)	0.523	57 (57.6)	137 (73.7)	0.022*
	AG	84 (29.2)	41 (27)		38 (38.4)	45 (24.2)	
	AA	8 (2.8)	2 (1.3)		4 (4)	4 (2.2)	
	MAF (A)	100 (17.4)	45 (14.8)	0.429	46 (23.2)	53 (14.2)	0.005*

Data are presented as absolute frequencies (%). AC: alcoholic cirrhosis; AWLD: alcoholics without liver disease. MAF: minor allele frequency. Some subjects could not be genotyped for technical reasons.

* Genotypic and allelic frequencies of the *RASGRF2* polymorphism showed a significantly different distribution in patients with ALC compared to those without ALD (*P* = 0.022 and 0.005, respectively).

**Table 2:** Genotypic and allelic frequencies of the *KRAS* and *RASGRF2* polymorphisms in patients with AA, AD and controls.

	AA (n = 112)	AD (n = 189)	*P*	Controls (n = 156)	P*	P**
*KRAS*						
TT	88 (79.3)	144 (76.6)		110 (71)		
GT	21 (18.9)	42 (22.3)	0.693	43 (27.7)	0.240	0.497
GG	2 (1.8)	2 (1.1)		2 (1.3)		
MAF (G)	25 (11.3)	46 (12.2)	0.591	47 (15.2)	0.125	0.237
*RASGRF2*						
GG	75 (73)	121 (65.4)		109 (71.7)		
AG	26 (25)	58 (31.4)	0.410	41 (27)	0.891	0.299
AA	2 (1.9)	6 (3.2)		2 (1.3)		
MAF (A)	30 (14.6)	70 (18.9)	0.241	45 (14.8)	0.847	0.216

Data are presented as absolute frequencies (%). AD: alcoholic dependence; AA: alcohol abuse. MAF: minor allele frequency. Some subjects could not be genotyped for technical reasons.

*P-value for the comparison of AA patients vs. controls.

**P-value for the comparison of AD patients vs. controls.

The genotypic distribution of the *KRAS* rs61764370 polymorphism showed no significant differences between groups (alcoholic patients vs. healthy controls, or AC patients vs. AWLD patients; Tables 1 and 2), apart from a nominally significant difference in genotypic but not allelic distribution between patients with AC and those without liver disease in the subgroup of patients with AD (supplementary Table 1).

In contrast, the genotypic distribution of the *RASGRF2* rs26907 polymorphism showed significant differences between AC patients and AWLD patients (*P* = 0.022, Table 1). Allelic analysis of this polymorphism also showed that the prevalence of the A allele was significantly higher among AC patients (23.2%) compared to AWLD patients (14.2%) (*P* = 0.005). These differences between AC and AWLD patients for this SNP were also found when the analysis was restricted to patients with AD, but not in the AA group (supplementary Table 1). No differences were found when comparing alcoholic patients with healthy controls or patients with AA vs. those with AD, and neither when comparing patients with AA or AD with controls (Table 2).

The analysis of the different models of inheritance for the RASGRF2 polymorphism and ALD is shown in Table 3 and the results of multivariable logistic regression analyses of the relationship between the *RASGRF2* polymorphism and ALD are presented in Table 4. After adjustment for age, allele A carriers (AA and GA genotypes combined) showed an OR of 2.33 (95% CI: 1.32–4.11; *P* = 0.002) for the presence of AC. We also performed this analysis in the subgroup of patients with AD, in which allele A carriers showed an OR of 3.34 (95% CI: 1.60–6.96; *P* = 0.001) for the presence of liver disease. According to the AIC scores, the dominant model for the A allele of this polymorphism was the most parsimonious model of inheritance on both analyses.

**Table 3:** Genotypic frequencies of the *RASGRF2* 113808 G>A (rs26907) polymorphism by disease status and model of inheritance for the A allele.

Model for the A allele	Alcoholic patients			Alcoholic patients with AD		
	AC patients	AWLD patients	*P*	AC patients	AWLD patients	*P*
General						
GG	59 (57.8)	137 (73.7)		28 (47.5)	93 (73.8)	
AG	39 (38.2)	45 (24.2)	0.022	28 (47.5)	30 (23.8)	0.002
AA	4 (4)	4 (2.2)		3 (5)	3 (2.4)	
Recessive						
AG + GG	98 (96)	182 (97.8)		56 (95)	123 (97.6)	
AA	4 (4)	4 (2.2)	0.382	3 (5)	3 (2.4)	0.385
Dominant						
GG	59 (57.8)	137 (73.7)		28 (47.5)	93 (73.8)	
AG + AA	43 (42.2)	49 (26.3)	0.006	31 (52.5)	33 (26.2)	<0.001

Data are presented as absolute frequencies (%). AC: alcoholic cirrhosis. AWLD: alcoholics without liver disease. Some subjects could not be genotyped for technical reasons.

**Table 4:** Results of the multivariable logistic regression analysis of the dominant model for the A allele of *RASGRF2* 113808 G>A (rs26907) polymorphism in cirrhotic patients.

			**Multivariable analysis**		
**Alcoholic patients**					
	AC patients	AWLD patients	OR	95% CI	*P*
Genotype					
GG	59 (57.8)	137 (73.7)	1	Reference	
AG + AA	43 (42.2)	49 (26.3)	2.33	1.32–4.11	0.002
Age	59 (11.8)	48.7 (11.3)	1.1	1.05–1.10	<0.001
**Alcoholic patients with AD**					
	AC patients	AWLD patients	OR	95% CI	*P*
Genotype					
GG	28 (47.5)	93 (73.8)	1	Reference	
AG + AA	31 (52.5)	33 (26.2)	3.1	1.50–6.20	0.001
Age	56.2 (10.8)	48.5 (9.6)	1.1	1.05–1.10	<0.001

Data are presented as absolute frequencies (%) for genotype and mean for age (SD). OR = odds ratio; CI = confidence interval; AC: alcoholic cirrhosis; AWLD: alcoholics without liver disease; AD: alcohol dependence. Some subjects could not be genotyped for technical reasons.

Post-hoc power calculations for our study showed a power of 16.4% for detecting differences between minor allele frequency of *RASGRF2* SNP between alcoholics and controls and a power of 77.9% for differences between alcoholics with AC and those without liver disease.

## Discussion

We found an association of the *RASGRF2* G>A polymorphism (rs26907) with the presence of AC in male alcoholic patients, but not with the presence of alcoholism itself (defined as AA or AD, according to DSM-IV criteria). Possession of the A allele of this SNP (genotype AA or AG) was associated with a higher risk of AC. Of note, this association remained significant among patients with AD, but not among those with AA. Conversely, the *KRAS* T>G polymorphism (rs61764370) showed no consistent association with the presence of ALD or alcohol use disorders. To our knowledge, this is the first study to describe a relationship between ALD and polymorphisms within the *RAS* gene family.

The association of *RASGRF2* polymorphisms and ALD is biologically plausible when we consider that RasGRF2 is involved in the inflammatory response through the activation of the MAPK/ERK pathway [15,31]. This pathway participates in the ethanol-induced inflammatory response mediated by Toll-like receptor 4 (TLR4) and nuclear factor kappa B (NF-κB) [32]. Additional experimental data regarding the MAPK/ERK pathway reinforce the potential association of RasGRF2 with ALD development. On the one hand, this pathway is involved in ALD development through negative regulation of hepatocyte differentiation and proliferation [33]. On the other hand, ERK activation by alcohol has been associated with macrophage stimulation [34] and ERK phosphorylation has been described in the peritoneal macrophages of ALD patients [35], which could be associated with an elevated inflammatory response. In light of these previous data and our own results, we could hypothesize that the presence of the A allele in the *RASGRF2* polymorphism may potentiate the RasGRF2-mediated ethanol-induced inflammatory response, leading to macrophage activation and an increased risk of liver fibrosis. Despite the lack of functional data for this polymorphism or information to perform an eQTL analysis, this hypothesis could explain the higher frequency of the A allele among AC patients compared to AWLD patients. Although subgroup analysis may be prone to type I error, the finding of a different risk in patients with AA or AD may reflect a higher level of alcohol consumption in patients with AD, which leads to an increased risk of AC development in the presence of the A allele.

In contrast, our negative result for the association with alcoholism (AA or AD) was unexpected, because previous findings have pointed towards an association of this polymorphism with alcohol use disorders [30]. This relationship was initially suggested by a GWAS showing an association between higher alcohol consumption and the presence of the G allele of rs26907 [11]. Reinforcing this finding, a haplotype containing the G allele of this polymorphism was associated with a different reward sensitivity related to alcohol exposure and, in adolescent males, with a higher number of drinking episodes [12]. A relationship between the *RASGRF2* G>A polymorphism and alcoholism itself is also biologically plausible. RasGRF2 can activate the MAPK/ERK pathway, which is involved in neurotransmission (especially through dopamine receptors and transporters) and thus potentially associated with reward mechanisms in alcoholism [36,37]. In mouse models, ethanol administration increased ERK activity in the *nucleus accumbens*, and inhibition of ERK activity influenced ethanol self-administration [38,39]. In addition, *RASGRF2* knockout mice showed decreased alcohol consumption owing to a diminished neuronal excitability, which was mediated not only by dopaminergic pathways [12], but also by a decrease in noradrenergic activation [40].

Given this background, it is difficult to reconcile the previous findings showing an association of the G allele of the *RASGRF2* SNP with higher alcohol intake and our own data showing an association of the A allele with ALD, particularly in patients with dependence. In this context, we have to consider the differences between the studies. Our large cohort of alcoholic patients was comprised of individuals with AA or AD. In contrast, previous works [12,13,40] analyzed patients with different alcohol-related phenotypes. Thus, our study is the first to explore the association of this polymorphism among patients with a diagnosis of alcohol-related disorders. Although we did not found an association with this SNP, further association studies will be necessary to clarify this relationship. Furthermore, our Spanish population may have a different genetic background compared to sample populations in other studies. This makes plausible the hypothesis that the discordant results may be due to a different pattern of linkage disequilibrium with other SNPs within this gene.

Regarding *KRAS* 3377 T>G (rs61764370) polymorphism, we did not find a consistent association of this SNP with ALD or alcoholism and we only observed, in the subgroup of patients with AD, a nominally significant higher frequency of the GT genotype in patients with AC when compared with those without liver disease. Although this result may be a false positive, we cannot dismiss the possibility that this gene plays a role in alcoholism, considering previous results from animal models [18]. Furthermore, this polymorphism is a target of the *let7-e* miRNA, which is potentially involved in both alcoholism and ALD development [20,41,42].

Despite the fact that we have controlled several potential confounders that could affect the validity of our findings, like addiction to other drugs, major psychiatric disorders, and age; we have to acknowledge several limitations of our work, such as the lack of detailed information regarding alcohol consumption levels. In addition, although we have limited the risk of population stratification, this bias is still possible. Another of the main limitations of our work is that our results can only be extrapolated to male subjects. The small number of patients with the AA genotype of the *RASGRF2* polymorphism makes it difficult to completely discard an additive model of inheritance for this SNP. Finally, the possibility of a type I error is an important concern in genetic studies, particularly when subgroup analysis are performed, and therefore our results should be interpreted with caution until they are independently replicated.

## Conclusions

To the best of our knowledge, our genetic association study represents the first to show an association of the *RASGRF2* G>A (rs26907) allelic variant with alcoholic liver cirrhosis in men, which was restricted to patients with AD. However, we did not find that this polymorphism was associated with alcohol use disorders. Our findings suggest the potential relevance of the *RAS* gene family in alcoholism and, more specifically, in ALD development. These results are of particular interest given previous findings linking this SNP to alcoholism. Further investigations are needed to clarify the role of these genetic variants in alcohol-related diseases.

## Supporting Information

S1 Table Supplementary Table 1. Genotypic and allelic frequencies of the *KRAS* and *RASGRF2* polymorphisms in patients with and without liver disease according to the presence of alcohol abuse or dependence.
(DOCX)

S2 Table Supplementary database. Clinical and genotypic data from all included patients.
(XLS)

## References

[1] Prescott CA, Kendler KS. Genetic and environmental contributions to alcohol abuse and dependence in a population-based sample of male twins. Am J Psychiatry. 1999;156: 34–40. 10.1176/ajp.156.1.34 9892295

[2] Reed T, Page WF, Viken RJ, Christian JC. Genetic predisposition to organ-specific endpoints of alcoholism. Alcohol Clin Exp Res. 1996;20: 1528–1533. 8986199 10.1111/j.1530-0277.1996.tb01695.x

[3] Köhnke MD. Approach to the genetics of alcoholism: a review based on pathophysiology. Biochem Pharmacol. 2008;75: 160–177. 10.1016/j.bcp.2007.06.021 17669369

[4] Tóth R, Fiatal S, Petrovski B, McKee M, Adány R. Combined effect of ADH1B RS1229984, RS2066702 and ADH1C RS1693482/ RS698 alleles on alcoholism and chronic liver diseases. Dis Markers. 2011;31: 267–277. 10.3233/DMA-2011-0828 22048268 PMC3826929

[5] Marcos M, Pastor I, Chamorro A-J, Ciria-Abad S, González-Sarmiento R, Laso F-J. Meta-analysis: glutathione-S-transferase allelic variants are associated with alcoholic liver disease. Aliment Pharmacol Ther. 2011;34: 1159–1172. 10.1111/j.1365-2036.2011.04862.x 21967547

[6] Gonzalez-Quintela A, Campos J, Loidi L, Quinteiro C, Perez L-F, Gude F. Serum TNF-alpha levels in relation to alcohol consumption and common TNF gene polymorphisms. Alcohol Fayettev N. 2008;42: 513–518. 10.1016/j.alcohol.2008.04.008 18579335

[7] Marcos M, Gómez-Munuera M, Pastor I, González-Sarmiento R, Laso F-J. Tumor necrosis factor polymorphisms and alcoholic liver disease: a HuGE review and meta-analysis. Am J Epidemiol. 2009;170: 948–956. 10.1093/aje/kwp236 19755636

[8] Zuo L, Tan Y, Zhang X, Wang X, Krystal J, Tabakoff B, et al A New Genomewide Association Meta-Analysis of Alcohol Dependence. Alcohol Clin Exp Res. 2015;39: 1388–1395. 10.1111/acer.12786 26173551 PMC5587504

[9] Barbacid M. ras genes. Annu Rev Biochem. 1987;56: 779–827. 10.1146/annurev.bi.56.070187.004023 3304147

[10] Coste I, Le Corf K, Kfoury A, Hmitou I, Druillennec S, Hainaut P, et al Dual function of MyD88 in RAS signaling and inflammation, leading to mouse and human cell transformation. J Clin Invest. 2010;120: 3663–3667. 10.1172/JCI42771 20941850 PMC2947228

[11] Schumann G, Coin LJ, Lourdusamy A, Charoen P, Berger KH, Stacey D, et al Genome-wide association and genetic functional studies identify autism susceptibility candidate 2 gene (AUTS2) in the regulation of alcohol consumption. Proc Natl Acad Sci U S A. 2011;108: 7119–7124. 10.1073/pnas.1017288108 21471458 PMC3084048

[12] Stacey D, Bilbao A, Maroteaux M, Jia T, Easton AC, Longueville S, et al RASGRF2 regulates alcohol-induced reinforcement by influencing mesolimbic dopamine neuron activity and dopamine release. Proc Natl Acad Sci U S A. 2012;109: 21128–21133. 10.1073/pnas.1211844110 23223532 PMC3529066

[13] Schwechter B, Rosenmund C, Tolias KF. RasGRF2 Rac-GEF activity couples NMDA receptor calcium flux to enhanced synaptic transmission. Proc Natl Acad Sci U S A. 2013;110: 14462–14467. 10.1073/pnas.1304340110 23940355 PMC3761609

[14] Tian X, Gotoh T, Tsuji K, Lo EH, Huang S, Feig LA. Developmentally regulated role for Ras-GRFs in coupling NMDA glutamate receptors to Ras, Erk and CREB. EMBO J. 2004;23: 1567–1575. 10.1038/sj.emboj.7600151 15029245 PMC391062

[15] Wang Q, Siminovitch KA, Downey GP, McCulloch CA. Ras-guanine-nucleotide-releasing factors 1 and 2 interact with PLCγ at focal adhesions to enable IL-1-induced Ca(2+) signalling, ERK activation and MMP-3 expression. Biochem J. 2013;449: 771–782. 10.1042/BJ20121170 23145787

[16] Karachaliou N, Mayo C, Costa C, Magrí I, Gimenez-Capitan A, Molina-Vila MA, et al KRAS mutations in lung cancer. Clin Lung Cancer. 2013;14: 205–214. 10.1016/j.cllc.2012.09.007 23122493

[17] Pilarski R, Patel DA, Weitzel J, McVeigh T, Dorairaj JJ, Heneghan HM, et al The KRAS-variant is associated with risk of developing double primary breast and ovarian cancer. PloS One. 2012;7: e37891 10.1371/journal.pone.0037891 22662244 PMC3360659

[18] Repunte-Canonigo V, van der Stap LD, Chen J, Sabino V, Wagner U, Zorrilla EP, et al Genome-wide gene expression analysis identifies K-ras as a regulator of alcohol intake. Brain Res. 2010;1339: 1–10. 10.1016/j.brainres.2010.03.063 20388501 PMC2925131

[19] Johnson C, Francis H, Glaser S, Han Y, Venter J, Liu C. 930 Regulation of Hepatic Stellate Cell Activation by MicroRNA Let-7 Family During Alcoholic Liver Injury.

[20] Lewohl JM, Nunez YO, Dodd PR, Tiwari GR, Harris RA, Mayfield RD. Up-regulation of microRNAs in brain of human alcoholics. Alcohol Clin Exp Res. 2011;35: 1928–1937. 10.1111/j.1530-0277.2011.01544.x 21651580 PMC3170679

[21] Gori M, Arciello M, Balsano C. MicroRNAs in nonalcoholic fatty liver disease: novel biomarkers and prognostic tools during the transition from steatosis to hepatocarcinoma. BioMed Res Int. 2014;2014: 741465 10.1155/2014/741465 24745023 PMC3972908

[22] American Psychiatric Association. Diagnostic and statistical manual of mental disorders (4th ed., text rev.). Wasington: American Psychiatric Publishing; 2000.

[23] Marcos M, Pastor I, González-Sarmiento R, Laso FJ. Interleukin-10 gene polymorphism is associated with alcoholism but not with alcoholic liver disease. Alcohol Alcohol Oxf Oxfs. 2008;43: 523–528. 10.1093/alcalc/agn026 18436572

[24] Marcos M, Pastor I, González-Sarmiento R, Laso F-J. A new genetic variant involved in genetic susceptibility to alcoholic liver cirrhosis: -330T>G polymorphism of the interleukin-2 gene. Eur J Gastroenterol Hepatol. 2008;20: 855–859. 10.1097/MEG.0b013e3282fd0db1 18794598

[25] Posada D, Buckley TR. Model selection and model averaging in phylogenetics: advantages of akaike information criterion and bayesian approaches over likelihood ratio tests. Syst Biol. 2004;53: 793–808. 10.1080/10635150490522304 15545256

[26] Dupont WD, Plummer WD. Power and sample size calculations for studies involving linear regression. Control Clin Trials. 1998;19: 589–601. 9875838 10.1016/s0197-2456(98)00037-3

[27] Kane SP. Post. ClinCalc: http://clincalc.com/stats/power.aspx. Updated October 24, 2015. Accessed October 16, 2016.

[28] Caiola E, Rulli E, Fruscio R, Buda A, Broggini M, Marabese M. KRas-LCS6 polymorphism does not impact on outcomes in ovarian cancer. Am J Cancer Res. 2012;2: 298–308. 22679560 PMC3365808

[29] Sebio A, Paré L, Páez D, Salazar J, González A, Sala N, et al The LCS6 polymorphism in the binding site of let-7 microRNA to the KRAS 3’-untranslated region: its role in the efficacy of anti-EGFR-based therapy in metastatic colorectal cancer patients. Pharmacogenet Genomics. 2013;23: 142–147. 10.1097/FPC.0b013e32835d9b0b 23324806

[30] Roussotte FF, Gutman BA, Hibar DP, Jahanshad N, Madsen SK, Jack CR, et al A single nucleotide polymorphism associated with reduced alcohol intake in the RASGRF2 gene predicts larger cortical volumes but faster longitudinal ventricular expansion in the elderly. Front Aging Neurosci. 2013;5: 93 10.3389/fnagi.2013.00093 24409144 PMC3867747

[31] Kyriakis JM, Avruch J. Mammalian MAPK signal transduction pathways activated by stress and inflammation: a 10-year update. Physiol Rev. 2012;92: 689–737. 10.1152/physrev.00028.2011 22535895

[32] Mandrekar P, Szabo G. Signalling pathways in alcohol-induced liver inflammation. J Hepatol. 2009;50: 1258–1266. 10.1016/j.jhep.2009.03.007 19398236 PMC3342816

[33] Gao W, Zhou P, Ma X, Tschudy-Seney B, Chen J, Magner NL, et al Ethanol negatively regulates hepatic differentiation of hESC by inhibition of the MAPK/ERK signaling pathway in vitro. PloS One. 2014;9: e112698 10.1371/journal.pone.0112698 25393427 PMC4231066

[34] Shi L, Kishore R, McMullen MR, Nagy LE. Chronic ethanol increases lipopolysaccharide-stimulated Egr-1 expression in RAW 264.7 macrophages: contribution to enhanced tumor necrosis factor alpha production. J Biol Chem. 2002;277: 14777–14785. 10.1074/jbc.M108967200 11856733

[35] Tapia-Abellán A, Martínez-Esparza M, Ruiz-Alcaraz AJ, Hernández-Caselles T, Martínez-Pascual C, Miras-López M, et al The peritoneal macrophage inflammatory profile in cirrhosis depends on the alcoholic or hepatitis C viral etiology and is related to ERK phosphorylation. BMC Immunol. 2012;13: 42 10.1186/1471-2172-13-42 22866973 PMC3496568

[36] Girault J-A, Valjent E, Caboche J, Hervé D. ERK2: a logical AND gate critical for drug-induced plasticity? Curr Opin Pharmacol. 2007;7: 77–85. 10.1016/j.coph.2006.08.012 17085074

[37] Maiya R, Ponomarev I, Linse KD, Harris RA, Mayfield RD. Defining the dopamine transporter proteome by convergent biochemical and in silico analyses. Genes Brain Behav. 2007;6: 97–106. 10.1111/j.1601-183X.2006.00236.x 16643512

[38] Vinci S, Ibba F, Longoni R, Spina L, Spiga S, Acquas E. Acetaldehyde elicits ERK phosphorylation in the rat nucleus accumbens and extended amygdala. Synap N Y N. 2010;64: 916–927. 10.1002/syn.20811 20506324

[39] Faccidomo S, Besheer J, Stanford PC, Hodge CW. Increased operant responding for ethanol in male C57BL/6J mice: specific regulation by the ERK1/2, but not JNK, MAP kinase pathway. Psychopharmacology (Berl). 2009;204: 135–147. 19125235 10.1007/s00213-008-1444-9 PMC2845162

[40] Easton AC, Rotter A, Lourdusamy A, Desrivixères S, Fernández-Medarde A, Biermann T, et al Rasgrf2 controls noradrenergic involvement in the acute and subchronic effects of alcohol in the brain. Psychopharmacology (Berl). 2014;231: 4199–4209. 24737505 10.1007/s00213-014-3562-x

[41] Bala S, Marcos M, Kodys K, Csak T, Catalano D, Mandrekar P, et al Up-regulation of microRNA-155 in macrophages contributes to increased tumor necrosis factor {alpha} (TNF{alpha}) production via increased mRNA half-life in alcoholic liver disease. J Biol Chem. 2011;286: 1436–1444. 10.1074/jbc.M110.145870 21062749 PMC3020752

[42] Novo-Veleiro I, Calle C de la, Domínguez-Quibén S, Pastor I, Marcos M, Laso F-J. Prevalence of hepatitis C virus infection in alcoholic patients: cohort study and systematic review. Alcohol Alcohol Oxf Oxfs. 2013;48: 564–569. 10.1093/alcalc/agt044 23690232

