# Supplementary material for: A Single Nucleotide Polymorphism in the RASGRF2 Gene Is Associated with Alcoholic Liver Cirrhosis in Men
Source: PLoS One. 2016 Dec 19;11(12):e0168685. doi: 10.1371/journal.pone.0168685 (PMC5167392; doi:10.1371/journal.pone.0168685)
Supplement: S1 Table — Genotypic and allelic frequencies of the KRAS and RASGRF2 polymorphisms in patients with and without liver disease according to the presence of alcohol abuse or dependence. (DOCX) [file pone.0168685.s001.docx]

**Supplementary Table 1**

**Genotypic and allelic frequencies of the *KRAS* and *RASGRF2* polymorphisms in patients with and without liver disease according to the presence of alcohol abuse or dependence.**

|  | Patients with AA | | | Patients with AD | | |
| --- | --- | --- | --- | --- | --- | --- |
|  | ALC | AWLD | *P* | ALC | AWLD | *P* |
| *KRAS* |  |  |  |  |  |  |
| TT | 34 (79.1) | 54 (79.4) |  | 50 (83.3) | 94 (73.4) |  |
| GT | 8 (18.6) | 13 (19.1) | 0.946 | 8 (13.3) | 34 (26.6) | 0.014* |
| GG | 1 (2.3) | 1 (1.5) |  | 2 (3.3) | 0 (0) |  |
| MAF (G) | 10 (11.6) | 15 (11) | 0.965 | 12 (10) | 34 (13.3) | 0.135 |
| *RASGRF2* |  |  |  |  |  |  |
| GG | 31 (72.1) | 44 (73.3) |  | 28 (47.5) | 93 (73.8) |  |
| AG | 11 (25.6) | 15 (25) | 0.969 | 28 (47.5) | 30 (23.8) | 0.002** |
| AA | 1 (2.3) | 1 (1.7) |  | 3 (5.1) | 3 (2.4) |  |
| MAF (A) | 13 (15.1) | 17 (14.4) | 0.849 | 34 (28.8) | 36 (14.3) | <0.001** |

Data are presented as absolute frequencies (%). Some subjects could not be genotyped for technical reasons. AA: alcohol abuse. AD: alcohol dependence. ALC: alcoholics with liver cirrhosis. AWLD: alcoholics without liver disease. MAF: minor allele frequency.

* Genotypic frequencies of the *KRAS* polymorphism showed a significantly different distribution in dependent patients with ALC compared to those without ALD (*P* = 0.014).

** Genotypic and allelic frequencies of the *RASGRF2* polymorphism showed a significantly different distribution in dependent patients with ALC compared to those without ALD (*P* = 0.002 and <0.001, respectively).
